# Factors associated with financial risk due to health spending in Argentina

**DOI:** 10.1093/heapol/czae051

**Published:** 2024-06-21

**Authors:** Juan Marcelo Virdis, María Eugenia Elorza, Fernando Delbianco

**Affiliations:** Health Organisation, Policy and Economics (HOPE) research group, Centre for Primary Care and Health Services Research (FBMH), University of Manchester, Manchester M3 9PL, UK; Instituto de Investigaciones Económicas y Sociales del Sur (IIESS), Universidad Nacional del Sur-CONICET, San Andrés 800, Bahía Blanca 8000, Argentina; Instituto de Investigaciones Económicas y Sociales del Sur (IIESS), Universidad Nacional del Sur-CONICET, San Andrés 800, Bahía Blanca 8000, Argentina; Departamento de Economía, Universidad Nacional del Sur (UNS), San Andrés 800, Bahía Blanca 8000, Argentina; Departamento de Economía, Universidad Nacional del Sur (UNS), San Andrés 800, Bahía Blanca 8000, Argentina; Instituto de Matemática de Bahía Blanca (INMABB), Universidad Nacional del Sur-CONICET, Av. Leandro N. Alem 1253, Bahía Blanca 8000, Argentina

**Keywords:** Catastrophic health expenditure, socioeconomic characteristics, generalized ordered logit model

## Abstract

This article aims to assess the association between household demographic and socioeconomic characteristics and catastrophic health expenditure (CHE) in Argentina during 2017–2018. CHE was estimated as the proportion of household consumption capacity (using both income and total consumption in separate estimations) allocated for Out-of-Pocket (OOP) health expenditure. For assessing the determinants, we estimated a generalized ordered logit model using different intensities of CHE (10%, 15%, 20% and 25%) as the ordinal dependent variable, and socioeconomic, demographic and geographical variables as explanatory factors. We found that having members older than 65 years and with long-term difficulties increased the likelihood of incurring CHE. Additionally, having an economically inactive household head was identified as a factor that increases this probability. However, the research did not yield consistent results regarding the relationship between public and private health insurance and consumption capacity. Our results, along with the robustness checks, suggest that the magnitude of the coefficients for the household head characteristics could be exaggerated in studies that overlook the attributes of other household members. In addition, these results emphasize the significance of accounting for long-term difficulties and indicate that omitting this factor could overestimate the impact of members aged over 65.

**Key messages**

- Elderly individuals and those with long-term health issues were found to be at a higher risk of experiencing catastrophic health expenditure.
- No conclusive evidence was found that public or private insurance reduces catastrophic health expenditure.
- Using household expenditure as a proxy for household consumption capacity and relying on head of household characteristics as representative of the entire household may introduce bias in research on the determinants of catastrophic health expenditure.

## Introduction

The study of Catastrophic Health Expenditure (CHE) has gained significant global importance over the years. The United Nations includes the proportion of the population with CHE among the indicators used to measure the achievement of the Sustainable Development Goals (World Health Organization, 2023). The importance of studying CHE lies in the concerns expressed by various countries and the international community regarding household impoverishment caused by high expenditures in medical services (World Health Assembly 58, 2005, p. 2). Despite the exhaustive assessment of CHE in the literature, the proportion of people suffering from CHE worldwide has been constantly increasing between 2000 and 2019 (World Health Organization, 2023, p. 27).

CHE indicators are based on household payment capabilities and Out-of-Pocket (OOP) expenditure, which is defined as the payments made at the time of receiving medical care. From these variables, the population experiencing CHE is identified as that living in households where OOP made by all members exceeds a certain proportion of the payment capacity of that household. Although extensive research has been conducted on various topics related to CHE, there is no consensus on the proportion of the payment capacity at which OOP becomes catastrophic (Wagstaff *et al*., 2018). Hence, different proportions are often used to obtain robust results (Wagstaff and Doorslaer, 2003; Somkotra and Lagrada, 2009; Khan *et al*., 2017; Wagstaff *et al*., 2018).

There are also diverse ways of estimating household payment capacity. For instance, Xu (2005) proposed that OOP should be compared with income or net subsistence expenditures, which can be determined as: (1) food expenditure, (2) value of a basic basket or (3) an international poverty line. However, Wagstaff (2019) noted that this methodology is not useful for assessing the OOP impact on subsistence expenditures. The author suggests that impoverishing expenditure indicators should be considered, which are based on households whose net income after OOP falls below the cost of a basket of basic goods and services. The author believed that CHE should be evaluated according to the total expenditure or income of households.

From the identification of households whose members have incurred CHE, it is possible to explore factors that characterize them. Studying the relationship between socioeconomic and demographic aspects and CHE incidence provides relevant results for formulating public policy, as it assesses if there are income segments, age ranges or households without health insurance that are particularly vulnerable to CHE. Previous analyses in different countries have found that in addition to health-related factors, various socioeconomic and demographic variables are associated with a higher likelihood of CHE (for a systematic review of determinants of CHE, see Azzani *et al*., 2019).

Although Argentina has improved its CHE indicators (Varco *et al*., 2022; Virdis *et al*., 2022), some authors have observed that they are high compared with Latin America and the Caribbean (Perticara, 2008; Knaul *et al*., 2011; Wagstaff *et al*., 2018a). Therefore, studying the associated factors is particularly relevant. In this regard, certain investigations on the aforementioned country assessing the link between CHE and socioeconomic and demographic variables are based on data from the Argentine Household Expenditure Survey (ENGHo, for its initials in Spanish) (National Institute of Statistics and Censuses, 2020a). The World Health Organization (2006) analysed the period 1996/97 and found that the probability of incurring CHE was lower in households with members under 5 years of age, or with a household head who is employed, has completed secondary education or has private health insurance. Conversely, households with a higher likelihood of presenting CHE were characterized by a household head over 64 years of age, using medical services and belonging to an income quintile higher than the first. On the other hand, Knaul *et al*. (2011) revealed that the factors associated with households with a higher likelihood evidencing CHE were being located in an urban area, low income, elderly members, lack of health insurance and a total household size of two or fewer people.

The most recent academic works on the factors related to OOP in Argentina used data from the ENGHo for the 2012/13 period (Abeldaño, 2017). Regarding this survey, the existence of methodological problems in data collection has been documented (National Institute of Statistics and Censuses, 2016, p. 11), which suggests that the latest reliable estimates likely correspond to the 2004/05 period. It is worth noting that between that time and the present, the Argentine economy and the National Health System of Argentina have undergone significant changes. For instance, in the 2004/18 period, GDP increased by 55% (National Institute of Statistics and Censuses, 2016a; 2018). Moreover, the proportion of the population benefiting from social insurance grew substantially, moving from 37.5% to 47.5% as a result of public policies implemented in the pension system (Bertranou, 2007; National Institute of Statistics and Censuses, 2020). Based on 2017/18 data, other studies estimated the fraction of the Argentine population presenting CHE (Varco *et al*., 2022; Virdis *et al*., 2022). However, while the latter did not evaluate the associated factors, the former only analysed the relationship between CHE and the income quintile to which the household belongs.

Argentina has a fragmented health system in which 57% of the population is covered by a mandatory health insurance scheme, 5% by voluntary private health insurance and 2% by other public plans (Ministry of Health, 2018). Therefore, 36% of the population is not covered by any insurance but can access a set of hospitals, infirmaries and practices whose services are available to all Argentine population. These providers are run by any of the three levels of public government: local, provincial and national. Only a small percentage of these providers are owned by the national government, and as a result, decentralization might create differences in the healthcare services available in different jurisdictions (Cetrángolo, 2014). Although the entire Argentine population is covered by at least one of these sub-systems, severe problems in providing effective coverage have been reported in the literature (Rubinstein *et al*., 2018). In addition, previous research has associated this system with a pro-rich distribution of healthcare services (Palacios *et al*., 2020).

The results obtained in this paper contribute to the literature on health system performance in countries aiming to expand health insurance. This topic has been extensively studied in the literature (World Health Organization, 2004). In particular, it has been examined whether mandatory health insurance is an effective strategy towards universal coverage (Mathauer *et al*., 2011).

The objective of this study is to assess the association between household demographic and socioeconomic characteristics and CHE. Specifically, we analyse whether income, education, the presence of elderly individuals, gender and individuals suffering from long-term conditions are associated with the probability of experiencing CHE. To this end, Section ‘Data and methods’ describes the data and methodology used in this study. Section ‘Results’ details the obtained results, while Section ‘Conclusions and discussion’ discusses these findings.

## Data and methods

We used data from the latest version of the ENGHo conducted by INDEC. The ENGHo is a survey that aims to understand household expenditure structures and characterize the population using socioeconomic variables (National Institute of Statistics and Censuses, 2020a). It was carried out between 2017 and 2018 on a sample designed in a three-stage procedure on households in localities with 2000 inhabitants or more^1^. The sample consisted of 44 922 households from which 21 547 responses were obtained, covering 68 725 inhabitants. The difference in the number of households and responses is due to non-eligibility of the household (6152), absence of respondents (4697), rejection of the survey (7483), rejection of some parts of the survey (4145) and other causes (727) (National Institute of Statistics and Censuses, 2020b). Each of the households was associated with expansion factors that allow adjusting the statistical measures for non-response, ineligible housing and calibration by known benchmarks or total populations (National Institute of Statistics and Censuses, 2020b). The databases were extracted from the official website (National Institute of Statistics and Censuses, 2020c).

OOP determinants were assessed by means of regressions in which the dependent variable was defined as $ca{t^z} = \left\{ {cat_1^z, \ldots ,cat_i^z, \ldots ,cat_n^z} \right\}$ for the *i*th *household*. This variable identifies households that have incurred CHE and was estimated from *gth_i_* and *gbs_i_* which denote the total expenditure and OOP for the *i*th household, respectively. The variable *cat^z^_i_* is equal to 1 if for the *i*th household *gbs_i_/gth_i_ > z*, and it is 0 in the rest of the cases, taking as limits *z *= {10%;15%;20%;25%}. The set *z* was chosen based on the limits established by the WHO to measure CHE (10% and 25%) (World Health Organization, 2023), between which two intermediate limits were added. The OOP variable was defined as the expenses incurred at the time of receiving health services (Xu, 2005; OECD, Eurostat, World Health Organization, 2017, p. 178). To calculate this, the data were obtained from the ‘Consumer Expenditure Division 6’, an item of the ENGHo corresponding to health expenditure. It groups the expenses for: pharmaceutical products, first aid items, therapeutic devices and equipment (and their repairs), medical consultations, dental services, auxiliary services for outpatients, admissions, surgeries and deliveries and health-related insurances. Following its definition, the OOP variable was determined as the ‘Consumer Expenditure Division 6’ minus the expenditure on health insurance (group 64 within the mentioned division).

Although the econometric models used lack a theoretical framework that defines a priori the explanatory variables to be included, the chosen variables align with suggestions found in the related literature (Xu *et al*., 2003; Azzani *et al*., 2019). Consequently, the explanatory variables used in the regressions are: proportion of members over 65 years of age; household head over 65 years of age; household size (number of members); household size squared; proportion of members with long-term difficulties^2^; proportion of members under 5 years of age; female household head^3^ (base: male); proportion of members covered by Comprehensive Medical Care Program (PAMI)^4^, non-profit mandatory health insurance, for-profit mandatory health insurance^5^ or voluntary health insurance; second, third, fourth and fifth quintile of expenditure per adult equivalent (only on specification I; base: first quintile); second, third, fourth and fifth quintile of income per adult equivalent (only on specification II; base: first quintile); unemployed or inactive household head (base: employed), low, medium, high and very high educational environment (base: very low); household located in Pampeana, Northwest, Northeast, Cuyo or Patagonia region (base: Greater Buenos Aires)^6^. These variables were chosen considering the works mentioned in Section ‘Data and methods’ that assessed determinants of OOP, to which was added the presence of a household head with long-term difficulties based on the survey by Azzani *et al*. (2019).

Household consumption capacity was included as an explanatory variable in two ways: expenditure quintile per adult equivalent and income quintile per adult equivalent. The adult equivalent is a measure used to compare the nutritional requirements of people of different gender and age. While this concept is primarily used in estimates related to food consumption, adjusting income to adult equivalent helps mitigate the bias of assuming that all household members have the same consumption requirements to achieve equal utility. Regarding consumption capacity, two different variables were considered due to the advantages and disadvantages of each. On the one hand, total expenditure has been pointed out as a more reliable variable in household surveys, as income tends to be a more sensitive dimension for household members, and in cases of income from agricultural or family businesses, it is challenging to measure (Deaton, 2018, p. 146). On the other, the income variable avoids the double causality problem that would be created if, in the face of a health event, OOP does not fully displace the consumption of other goods. These households will (ceteris paribus) have higher total expenditure and, therefore, a greater likelihood of belonging to a higher expenditure quintile. In this way, OOP, which contributes to constructing the dependent variable, would impact the expenditure quintile per adult equivalent, which is part of the explanatory variables. From now on, specification I refers to estimates that comprise the expenditure quintile per adult equivalent, and specification II represents those of the income quintile per adult equivalent. The rest of the covariables remain the same in both specifications.

To make the estimates, a Generalized Ordered Logit model was applied. Logit regressions are typically used when the dependent variable is binary (Long, 1997, p.192). Since $p\left( {y = 1} \right)$ is the probability of obtaining ${y_i} = 1$ when observing ${X_i}$, the parameters $\beta $ of the following model were estimated:


$$ln\frac{{p\left( {{y_i} = 1} \right)}}{{p\left( {{y_i} = 1} \right) - 1}} = {X_i}\beta $$

$${p_i}\left( {{y_i} = 1} \right) = g\left( {{X_i}\beta } \right) = \frac{{{e^{{X_i}\beta }}}}{{1 + {e^{{X_i}\beta }}}}$$

If the estimate includes an intercept ${\beta _0} \in \beta $, we assume that ${x_{i0}} = 1\,\forall \,i$.

Specifically, in the assessment of households that have incurred CHE, although the dependent variable is binary, different thresholds can be applied for their classification (in this article, we used four: *z *= {10%;15%;20%;25%}). Thus, it could be verified that $cat_i^{10\% } = 1$ and $cat_i^{15\% } = 0$, where $cat_i^z$ denotes if household $i$ incurred in CHE using $z$ as limit. As indicated in Section ‘Introduction’, there is no consensus on the threshold at which OOP becomes catastrophic for households. For this reason, the Generalized Ordered Logit model (Williams, 2016) was employed, which allows including various thresholds. This model should be applied when the assumption of parallel slopes in the Ordered Logit model is not met. The non-fulfilment of this assumption was assessed through the Brant test, the results of which can be seen in the supplementary material. From the variables $ca{t^z}$, the categorical one was constructed as


$$y_{i} = \begin{cases} 1\,\,\,\, \text{ if } cat_{i}^{10\%} = 0 \\ 2\,\,\,\, \text{ if } cat_{i}^{10\%} = 1 \,\,\text{ and } cat_{i}^{15\%} = 0 \\ 3\,\,\,\, \text{ if } cat_{i}^{15\%} = 1 \,\,\text{ and } cat_{i}^{20\%} = 0 \\ 4\,\,\,\, \text{ if } cat_{i}^{20\%} = 1 \,\,\text{ and } cat_{i}^{25\%} = 0 \\ 5\,\,\,\, \text{ if } cat_{i}^{25\%} = 1 \end{cases}$$

The following probabilities can be constructed from the defined categories:


$$p\left( {{y_i} = 1} \right) = 1 - g\left( {{X_i}{\beta _1}} \right)\\[-3pt]$$

$$p\left( {{y_i} = j} \right) = g\left( {{X_i}{\beta _{j - 1}}} \right) - g\left( {{X_i}{\beta _j}} \right){\ }for{\ }j = 2,3,4 \\[-3pt] $$

$$p\left( {{y_i} = 5} \right) = g\left( {{X_i}{\beta _4}} \right){\ }$$

From the estimation, using sample weights, four sets of coefficients were derived. Each set of coefficients represents the odds ratio between the lower and higher categories of CHE within each threshold. In the subsequent sections, the estimation related to thresholds between the lower category $\underline{z}\%$ and the higher category $\bar z\% $ is denoted as $cat_{\underline{z}}^{\bar z}$. Therefore, the estimations are denoted as $cat_0^{10}$, $cat_{10}^{15}$, $cat_{15}^{20}$ and $cat_{20}^{25}$. We present the coefficients of the main results, along with their standard deviations and *P*-values. Additionally, tables demonstrating evidence of the violation of parallel trend assumptions through the Brant test (Brant, 1990) and other estimations serving as robustness checks are provided in supplementary material. The regressions models were performed using the gologit2 package for Stata (Williams, 2006).

## Results

Table 1 presents the descriptive statistics of the variables utilized in this study. It is evident that the majority of households do not register positive OOP, given that the median stands at zero. In addition, households in the sample allocate 4.53% of their total expenditure and 3.4% of their total income to OOP. The proportion of members facing long-term difficulties or those aged over 65 is low in most households, as indicated by the 75th percentile being zero for the former and 0.2 for the latter. The ratio of households incurring CHE is 0.112 for $z = 10\% $, 0.069 for $z = 15\% $, 0.045 for $z = 20\% $ and 0.03 for $z = 25\% $. A considerable number of individuals benefit from non-profit mandatory insurance, with more that 25% of households being completely covered by this insurance. Lastly, a medium educational environment characterizes the majority of households.

**Table 1.** Descriptive statistics of households in the sample

Statistic	Mean	St. Dev.	Pctl(25)	Median	Pctl(75)
Per capita OOP^a^	29.733	105.047	0.000	0.000	20.648
Per capita expenditure^a^	655.707	673.374	251.429	446.908	814.954
Per capita income^a^	874.877	1157.484	351.420	609.036	1068.496
Size	3.190	1.852	2	3	4
Prop. of members with LT difficulties	0.112	0.251	0.000	0.000	0.000
Prop of members older than 65 YOA	0.179	0.341	0.000	0.000	0.200
Head older than 65 YOA (0 = No; 1 = yes)	0.230	0.421	No	No	No
Prop. of households with CHE*^z^* (0 = No; 1 = yes) *z *= 10%	0.112	0.315	No	No	No
*z *= 15%	0.069	0.254	No	No	No
*z *= 20%	0.045	0.208	No	No	No
*z *= 25%	0.030	0.172	No	No	No
Prop. covered by PAMI	0.161	0.326	0.000	0.000	0.000
Non-profit mandatory insurance	0.453	0.444	0.000	0.333	1.000
For-profit mandatory insurance	0.043	0.188	0.000	0.000	0.000
Voluntary insurance	0.049	0.202	0.000	0.000	0.000
Economic status of the household head (0 = No; 1 = yes) Employed	0.648	0.478	No	Yes	Yes
Unemployed	0.035	0.185	No	No	No
Inactive	0.317	0.465	No	No	Yes
Household educational environment Very low	0.091	0.288			
Low	0.37	0.483	Low^b^	Medium^b^	Medium^b^
Medium	0.323	0.467			
High	0.129	0.335			
Very high	0.087	0.282			

a PPP dollars (OECD, 2023). Abbreviations: OOP = Out-of-pocket expenditure; YOA = Years of Age; LT = Long Term; CHE = Catastrophic Health Expenditure.

b Only one set of position measures is reported for ‘Very Low’, ‘Low’, ‘Medium’, ‘High’ and ‘Very High’, as they represent categories of a single ordinal variable related to educational environment.

Tables 2 and 3 display the results for specifications I and II, respectively. Different coefficients related to household composition were found to be statistically significant. In specification I, the proportion of members above 65 years of age exhibited odds ratios (OR) between 1.69 and 2.56 ($PV < 0.05$ in $cat_0^{10}$ and $cat_{10}^{15}$). In specification II, the OR were slightly higher, with values ranging between 2.05 and 2.84 ($PV < 0.05$ in $cat_0^{10}$, $cat_{10}^{15}$, and $cat_{15}^{20}$). These results associate the presence of people older than 65 with a higher probability of CHE. Regarding household size, the results indicate the existence of scale economies, as its coefficient was estimated to be between 1.21 and 1.81, and its squared version between 0.94 and 0.97. These coefficients were statistically significant in specification I, and for $cat_0^{10}$ and $cat_{10}^{15}$ in specification II ($PV < 0.05$). For members with long-term difficulties, OR values between 2.61 and 3.07 were identified. These coefficients were statistically significant across all specifications. The coefficients related to a female household head were greater than one, but only for $cat_0^{10}$ were they found to be statistically significant.

**Table 2.** Odds ratios from the generalized ordered logit model: household characteristics associated with CHE (specification I)

	0% and 10%		10% and 15%		15% and 20%		20% and 25%	
	OR	SE	OR	SE	OR	SE	OR	SE
Household composition								
Prop. of members above 65 YOA	2.556***	(0.549)	2.257***	(0.577)	1.741*	(0.551)	1.688	(0.639)
Household head above 65 YOA	0.869	(0.144)	1.171	(0.227)	1.495*	(0.348)	1.621*	(0.468)
Household size	1.674***	(0.141)	1.807***	(0.191)	1.586***	(0.214)	1.658***	(0.277)
Household size squared	0.944***	(0.0102)	0.938***	(0.0121)	0.952***	(0.0162)	0.945***	(0.0199)
Prop. of member with long term difficulties	2.732***	(0.310)	2.701***	(0.344)	2.883***	(0.434)	3.067***	(0.582)
Prop. of members under 5 YOA	0.788***	(0.0725)	0.791**	(0.0931)	0.833	(0.142)	0.887	(0.218)
Female household head (Base: male)	1.222***	(0.0845)	1.177*	(0.0999)	1.141	(0.117)	1.267*	(0.171)
Health insurance								
Prop. of members with PAMI	1.023	(0.141)	0.872	(0.146)	0.835	(0.163)	0.809	(0.178)
Prop. of members with non-profit mandatory insurance	1.009	(0.103)	0.771**	(0.0999)	0.777	(0.129)	0.788	(0.165)
Prop. of members with for-profit mandatory insurance	0.788	(0.146)	0.526**	(0.133)	0.450**	(0.144)	0.402*	(0.187)
Prop. of members with private insurance	0.748*	(0.116)	0.463***	(0.0982)	0.492***	(0.121)	0.371***	(0.104)
Observations	21 523							
	0% and 10%		10% and 15%		15% and 20%		20% and 25%	
	**Coef.**	**SE**	**Coef.**	**SE**	**Coef.**	**SE**	**Coef.**	**SE**
Income quintile per EA. Base category: first quintile								
Second quintile	1.854***	(0.247)	1.813***	(0.298)	1.539**	(0.328)	1.356	(0.357)
Third quintile	2.435***	(0.330)	2.682***	(0.438)	2.815***	(0.595)	2.560***	(0.687)
Fourth quintile	3.036***	(0.429)	3.127***	(0.528)	3.394***	(0.721)	2.734***	(0.745)
Fifth quintile	4.899***	(0.740)	5.549***	(1.000)	6.732***	(1.610)	6.423***	(1.954)
Household head economic status. Base category: employed								
Unemployed	1.624**	(0.313)	1.288	(0.316)	1.092	(0.331)	0.725	(0.263)
Inactive	1.467***	(0.139)	1.524***	(0.171)	1.671***	(0.222)	1.689***	(0.290)
Household educational environment. Base category: very low								
Low	0.939	(0.107)	0.882	(0.118)	0.858	(0.143)	0.939	(0.191)
Medium	0.729**	(0.0930)	0.631***	(0.0939)	0.649**	(0.122)	0.713	(0.164)
High	0.626***	(0.0955)	0.573***	(0.103)	0.495***	(0.113)	0.502**	(0.142)
Very high	0.556***	(0.0939)	0.551***	(0.108)	0.500***	(0.128)	0.416**	(0.142)
Constant term	0.0120***	(0.00297)	0.00705***	(0.00231)	0.00530***	(0.00213)	0.00316***	(0.00167)
Observations	21 523							

Note. Robust standard deviation in parentheses.

Abbreviations: EA = equivalent adult; OR = Odd Ratio; SE = Standard Error; YOA = Years of Age; PAMI = Comprehensive Medical Care Program.

* *P* < 0.1;

** *P* < 0.05;

*** *P* < 0.01.

**Table 3.** Odds ratios from the generalized ordered logit model: household characteristics associated with CHE (specification II)

	0% and 10%		10% and 15%		15% and 20%		20% and 25%	
	OR	SE	OR	SE	OR	SE	OR	SE
Household composition								
Prop. of members above 65 YOA	2.836***	(0.603)	2.732***	(0.694)	2.118**	(0.672)	2.054*	(0.758)
Household head above 65 YOA	0.842	(0.139)	1.121	(0.214)	1.479*	(0.337)	1.629*	(0.452)
Household size	1.451***	(0.120)	1.462***	(0.147)	1.215	(0.161)	1.210	(0.186)
Household size squared	0.950***	(0.00995)	0.950***	(0.0115)	0.969*	(0.0164)	0.966*	(0.0187)
Prop. of member with long term difficulties	2.681***	(0.298)	2.605***	(0.323)	2.797***	(0.407)	2.817***	(0.545)
Prop. of members under 5 YOA	0.813**	(0.0746)	0.801*	(0.0950)	0.828	(0.146)	0.853	(0.204)
Female household head (Base: male)	1.215***	(0.0829)	1.178*	(0.0993)	1.150	(0.119)	1.246	(0.170)
Health insurance								
Prop. of members with PAMI	1.098	(0.150)	0.985	(0.166)	0.940	(0.183)	0.943	(0.203)
Prop. of members with non-profit mandatory insurance	1.231**	(0.121)	1.061	(0.139)	1.087	(0.178)	1.145	(0.229)
Prop. of members with for-profit mandatory insurance	1.094	(0.201)	0.918	(0.230)	0.822	(0.260)	0.808	(0.367)
Prop. of members with private insurance	1.019	(0.154)	0.712	(0.152)	0.773	(0.190)	0.601*	(0.172)
Observations	21 523							
	0% and 10%		10% and 15%		15% and 20%		20% and 25%	
	**Coef.**	**SE**	**Coef.**	**SE**	**Coef.**	**SE**	**Coef.**	**SE**
Income quintile per EA. Base category: first quintile								
Second quintile	0.930	(0.109)	0.920	(0.134)	0.941	(0.168)	0.936	(0.210)
Third quintile	1.008	(0.125)	0.809	(0.130)	0.755	(0.138)	0.620**	(0.145)
Fourth quintile	1.234	(0.158)	1.124	(0.186)	1.159	(0.229)	0.953	(0.241)
Fifth quintile	1.168	(0.166)	0.880	(0.162)	0.916	(0.209)	0.766	(0.208)
Household head economic status. Base category: employed								
Unemployed	1.449**	(0.266)	1.076	(0.260)	0.947	(0.291)	0.638	(0.228)
Inactive	1.400***	(0.131)	1.431***	(0.160)	1.583***	(0.207)	1.656***	(0.278)
Household educational environment. Base category: very low								
Low	1.024	(0.117)	0.973	(0.130)	0.987	(0.157)	1.066	(0.204)
Medium	0.896	(0.113)	0.804	(0.118)	0.877	(0.157)	0.943	(0.206)
High	0.869	(0.130)	0.830	(0.146)	0.777	(0.173)	0.796	(0.215)
Very high	0.836	(0.139)	0.871	(0.169)	0.872	(0.222)	0.752	(0.250)
Constant term	0.0301***	(0.00729)	0.0216***	(0.00693)	0.0176***	(0.00681)	0.0118***	(0.00581)
Observations	21 523							

Note. Robust standard deviation in parentheses.

Abbreviations: EA = equivalent adult; OR = Odd Ratio; SE = Standard Error; YOA = Years of Age; PAMI = Comprehensive Medical Care Program.

* *P* < 0.1;

** *P* < 0.05;

*** *P* < 0.01.

In the case of economic inactivity of the household head, using an employed one as the base category, OR indicates that it is associated with an increase in the likelihood of incurring CHE in all estimates, with the OR between 1.47 and 1.69 in specification I, and between 1.4 and 1.66 in specification II ($PV < 0.01$ in all cases). As for the educational level, only statistically significant parameters were associated with a medium, high and very high in specification I.

Concerning consumption capacity, in specification I, the results evidence that households with a higher consumption capacity are more likely to incur CHE. The OR in $cat_0^{10}$ for expenditure quintiles 2, 3, 4 and 5 were 1.86, 2.44, 3.04, and 4.9, respectively, taking quintile one as base category. Statistical significance was verified for all the $z$ limits used and for all expenditure quintiles, except for quintile two in $cat_{20}^{25}$. For the income quintile per equivalent adult, part of specification II, the observed parameters are close to one and were mostly not significant.

Lastly, regarding health insurance, we found no definitive results concerning its impact on the probability of CHE. For voluntary insurance in specification I, the OR was determined to be between 0.37 and 0.75 ($PV < 0.05$ except for $cat_0^{10}$). However, the parameters estimated in specification II were not significant ($PV > 0.05$). For PAMI and non-profit mandatory work insurance, the results were inconsistent or non-significant. Of all the insurance types assessed, for-profit insurance was the only one associated with a decreased likelihood.

The supplementary material includes several robustness exercises. The results of different specifications of the ordinary Logit Model confirm an increased likelihood of CHE in households where members experience long-term difficulties or are over 65 years of age. Also, the head of household’s long-term difficulties as an explanatory variable was found to be statistically not significant.

## Conclusions and discussion

In this study, a set of determinants for financial risk due to OOP was identified, highlighting population groups in Argentina that experienced lesser financial protection against CHE in the years 2017 and 2018. The results were obtained from regressions of the Generalized Ordered Logit Model, representing a methodological contribution compared with previous studies conducted in Argentina. This is particularly relevant as the threshold at which CHE becomes catastrophic is currently a subject of discussion (Wagstaff *et al*., 2018). Notably, we found that some of the studied factors, such as having a female household head or a higher proportion of members under 5 years of age, exhibited varying degrees of statistical significance depending on the threshold used to estimate CHE. This approach has also been applied in other Low and Middle-Income countries, finding different results based on the chosen threshold (Buigut *et al*., 2015; Özgen Narcı *et al*., 2015).

The results obtained in relation to the presence of older adults in the household are consistent with studies that assessed the determinants of OOP or CHE in Argentina and other Latin-American countries (World Health Organization, 2006; Perticara, 2008; Knaul *et al*., 2011; Abeldaño, 2017; Maceira, 2018). Similarly, there is agreement that the employment situation has an impact on CHE (World Health Organization, 2006; Perticara, 2008). One of the novel results of this work was the positive link between the probability of OOP and the presence of a household head with long-term difficulties. This variable was not evaluated in the aforementioned works, because this aspect was not surveyed in versions of the ENGHo prior to 2017/18. However, the disability of the household head has been highlighted as a significant determinant in analyses carried out in other countries (Azzani *et al*., 2019; Saenz-Vela and Guzman-Giraldo, 2021).

It is worth noting that people aged 65 and above are associated with a higher probability of CHE, despite being the segment of the population with the highest levels of insurance coverage, nearly 100%. This might be explained by failures in the coverage provided by PAMI, causing people associated with this institution to still rely significantly on OOP expenses. Additionally, the existence of a fragmented system might cause people to access either PAMI or publicly owned providers, obtaining similar levels of coverage. If this is the case, significant cross-subsidies are occurring, and excessive financial pressure is being borne by these publicly owned providers. Moreover, this is consistent with the non-significant result associated with PAMI affiliation. Although more health state variables are required in the model to clarify the possibility of these coefficients capturing a part of health status, if the coefficients obtained in the model are accurate, the need for a different insurance scheme for the elderly, rather than a universal insurance covering the entire population, might be questioned. However, if the higher probability of CHE for people aged 65 and above is due to inefficacy or problems in the design of PAMI, having this targeted insurance might be a good way to channel policy intended to reduce OOP expenses. To elucidate these issues, more understanding of the characteristics of the elderly health care spending is needed. For instance, given that PAMI provides healthcare at zero cost except for pharmaceutical services, these services could be causing CHE.

In relation to consumption capacity, the results show a positive association between higher expenditure quintiles per adult equivalent and the probability of CHE. These findings imply, counterintuitively, that households with lower consumption capacity have greater financial protection. Although this was evidenced in previous studies (World Health Organization, 2006; Knaul *et al*., 2011; Abeldaño, 2017), it could be the result of simultaneity bias (Wooldridge, 2016, p. 504). It is worth noting that the dependent variable is a function of CHE and the ENGHo data are cross-sectional. Therefore, it was not possible to analyse whether for households incurring CHE the total observed expenditure corresponds to their consumption habits or has been increased because of occasional medical-health care expenses. The presence of this bias is consistent with the results of specification II, in which the variable that approximates the consumption capacity is the income quintile per adult equivalent, and the coefficients, in general, were not statistically significant. In addition, the results obtained could be affected by the potential bias derived from the way the consumption capacity is measured. The effect of the number of spending items and the recall time for the calculation of catastrophic spending has been discussed for some surveys (Lu *et al*., 2009). The impact on the CHE of these measurement errors in India has recently been estimated (Mohanty *et al*., 2023). In Argentina, the effect of these biases in the ENGho is unknown and further research is needed on this topic.

Regarding the health insurance variables, we did not find robust results suggesting a lower likelihood of incurring CHE. The causes of this could lie in endogeneity problems in the variables that identify the health insurance of household heads. The database only includes long-term difficulties as the health status variables for household members, omitting other factors that could simultaneously increase the likelihood of CHE and the incentives to obtain health insurance. However, a positive association between health insurance and the probability of OOP has been found in previous studies for Argentina (Knaul *et al*., 2011) and for other countries that included the health status of household members among their explanatory variables (Ekman, 2007; Li *et al*., 2013; 2014). Other authors have pointed out that this could be explained by the fact that access to medical consultations financed by health insurance entails an induced demand for services that must be financed by patients (Wang, 2009; Ang, 2010; Barros *et al*., 2011; Chaabouni and Abednnadher, 2014). Additionally, Fan *et al*. (2021) observed that the induced demand can be verified for medications. In addition, the Argentine Health System is composed of public providers accessible to the entire Argentine population. Consequently, insurance coverage can be viewed as an improvement over the previous coverage available in the public sector. Further research is needed to determine whether significant differences exist between public providers and social insurance coverage. If differences are minimal or non-existent, non-significant coefficients should be found for insurance coverage.

Finally, the interpretation of the results of this work should consider the following limitations. First, the CHE data are cross-sectional, and strategies for distributing healthcare costs over different consumption periods were not evaluated. These strategies include indebtedness and asset sales, and their omission may overestimate the CHE incidence (Flores *et al*., 2008). Moreover, it is not clear whether consumption capacity should be measured in terms of income or expenditure; this has been related to individual behaviour towards financial shock due to health, which is hard to infer from available data (Wagstaff, 2019). Second, CHE does not include expenses derived from medical care such as transportation costs to clinics and hospitals, or indirect costs such as loss of income due to illness. These factors could significantly increase OOP incidence (Nguyen *et al*., 2013; Weraphong *et al*., 2013; Mullerpattan *et al*., 2019). Third, the databases used do not contain information on the health status of household members, which may be a relevant explanatory variable for CHE according to previous research in other countries (Su *et al*., 2006; Gotsadze *et al*., 2009; Somkotra and Lagrada, 2009). Lastly, unlike the 2004/05 version of the ENGHo, the sample considered in this article corresponding to the 2017/18 version does not include the rural population of Argentina. For this reason, it was not possible to evaluate changes in relation to the results of Knaul *et al*. (2011), in which the urban population was associated with a higher probability of OOP. On the other hand, as a methodological limitation, we must keep in mind the parameterization assumption made by the generalized ordered logit. Although a constant effect of passing between levels is not assumed, it is assumed that these effects are constant and parameterizable. Possible extensions of the work could consider non-parametric estimation methodologies, which, although they make the interpretation of the effects more difficult, can give an idea of the degree of fit of the data to the specified model.

In conclusion, despite the mentioned limitations, this study has provided valuable insights into two key determinants of CHE. First, our estimations, as presented in the results section, raise questions about the size of the effect on people over 65 years, as highlighted in previous studies. Since our results demonstrate significant coefficients for members suffering from long-term difficulties, and this often correlates with age, omitting the long-term difficulties variable can lead to an overestimated coefficient for people over 65 years of age. Secondly, a parallel conclusion can be inferred both from the characteristics of household members and those of the household head. A household head over 65 years of age positively correlates as a variable with the proportion of members over 65 years of age. Similarly, a household head suffering from long-term difficulties correlates with the proportion of members experiencing long-term difficulties. From the estimates in Tables 2 and 3, it is evident that a household head above 65 years of age exhibits lower (and non-significant) coefficients than the proportion of members over 65 years of age. Furthermore, in robustness exercises, as provided in the supplementary material, a household head with long-term difficulties shows nearly non-significant coefficients close to one, while the proportion of members demonstrates highly significant coefficients. In this context, a household head’s characteristics alone can lead to overestimation of coefficients.

These findings bear significant policy implications. While the household head is undoubtedly a vital member of the household, our results indicate that relying solely on their characteristics may not fully capture the nuances of a household. It is suggested that variables encompassing all members could offer more informative insights. Additionally, the current exclusive provision of universal coverage for the elderly by PAMI raises questions about the adequacy of such an approach. Consideration should be given to expanding universal coverage to include individuals facing long-term difficulties, as they appear to bear the most significant financial burden from OOP expenses. This broader approach would likely contribute to a more equitable and effective policy framework. As a future research agenda, it is necessary to delve deeper into understanding which specific long-term difficulties are linked to CHE. Furthermore, additional research is needed to assess the extent to which long-term difficulties are associated with income and OOP expenses. This issue is relevant to inform public policy decisions regarding healthcare coverage and income support measures. While this analysis is based on a sample from one country, its implications may hold importance for others as well.

## Supplementary Material

czae051_Supp

## Data Availability

The data underlying this article are available in the supplementary files. The datasets were derived from sources in the public domain: https://www.indec.gob.ar/indec/web/Institucional-Indec-BasesDeDatos-4.

## Footnotes

1. For more details on the sample design, see National Institute of Statistics and Censuses (2020b).

2. Household members are identified as having long-term difficulties if they suffer from any of the following: walking, going up or down stairs, grabbing objects and/or opening containers, seeing, even with glasses, hearing, even with a hearing aid, speaking, communicating or conversing, bathing, dressing or eating, learning simple things or paying attention or relating and/or working due to a mental issue.

3. In the ENGHo, the household head is the person considered as such by the other members of the household. In each, there is only one head, so there are as many heads as households in the database (INDEC no date).

4. PAMI is a public mandatory insurance that has as its main objective to finance health care services for the elderly.

5. Non-profit and for-profit mandatory insurance are typically provided by the workers’ union and can be extended to cover the entire family group.

6. These regions differ greatly in both population and density, which might affect healthcare accessibility, ranging from 3218.45 people/km² in Greater Buenos Aires to 1.25 people/km² in Patagonia. Pampeana stands at 16.75 people/km², Northwest at 8.75 people/km², Northeast at 12.50 people/km² and Cuyo at 9.12 people/km², averaging 5.25 people/km² across all regions.

## Reflexivity statement

The study was conducted by a team of three researchers from Latin America, with one female member. It emphasizes the importance of recognizing gender and regional inclusivity.
